# Quiescent Elongation of α-Synuclein Pre-form Fibrils Under Different Solution Conditions

**DOI:** 10.3389/fnins.2022.902077

**Published:** 2022-05-25

**Authors:** Hengxu Mao, Yongyi Ye, Xiang Sun, Chen Qian, Baoyan Wang, Linghai Xie, Shizhong Zhang

**Affiliations:** ^1^Guangdong Provincial Key Laboratory on Brain Function Repair and Regeneration, Department of Neurosurgery, The Engineering Technology Research Center of Education Ministry of China, Zhujiang Hospital, Southern Medical University, Guangzhou, China; ^2^Department of Neurosurgery, The Second Affiliated Hospital of Guangzhou Medical University, Guangzhou, China

**Keywords:** α-synuclein, aggregates, PFFs, PD, fibrillation, synucleinopathy, THT, prion-like diseases

## Abstract

The intracellular aggregation of α-synuclein in neurons/glia is considered to be a key step in the pathogenesis of synucleinopathy [including Parkinson’s disease (PD), dementia with Lewy body (DLB), multiple system atrophy (MSA), etc.]. Increasing evidence indicates that the initial pathological α-synuclein aggregates can replicate themselves and propagate in a “seeding” manner to multiple areas of the brain and even to peripheral tissue, which makes it the most important biomarker for the diagnosis of synucleinopathies in recent years. The amplification and propagation capabilities of α-synuclein aggregates are very similar to those of prion-like diseases, which are based on the inherent self-recruitment capabilities of existing misfolded proteins. *In vitro*, the rapid recruitment process can be reproduced in a simplified model by adding a small amount of α-synuclein pre-formed fibrils to the monomer solution as fibril seeds, which may partially reveal the properties of α-synuclein aggregates. In this study, we explored the elongation rate of α-synuclein pre-formed fibrils under a quiescent incubation condition (rather than shaking/agitating). By using the ThT fluorescence assay, we compared and quantified the elongation fluorescence curves to explore the factors that affect fibril elongation. These factors include proteins’ concentration, temperature, NaCl strength, SDS, temperature pretreatment, and so on. Our work further describes the elongation of α-synuclein fibrils under quiescent incubation conditions. This may have important implications for the *in vitro* amplification and preservation of α-synuclein aggregates to further understand the prion-like transmission mechanism of PD.

## Introduction

Synucleinopathies are common neurodegenerative diseases that include Parkinson’s disease (PD), multiple system atrophy (MSA), and dementia with LB (DLB). The pathological hallmark of these diseases is the intracellular aggregates of α-synuclein in the central nervous system, which are widely known as LB and LN (Spillantini et al., 1997). Alpha-synuclein is a 140-amino acid protein that is highly enriched in presynaptic nerve terminals and can pathologically fold into a β-sheet-rich structure that polymerizes into oligomers, fibrils, and aggregates. These misfolded proteins proved to be conformationally pathologic mainly due to the neurotoxicity form of oligomers/fibrils (rather than monomers), which will gradually damage the mitochondria, Hu et al. (2019), Wang et al. (2019) ubiquitin-proteasome systems, Lindersson et al. (2004) and autophagolysosomal systems (Lee et al., 2004), and finally leads to neurological dysfunction and cell death (Winner et al., 2011).

Increasing evidence shows that the injection of the pathological fibrils isolated from patients’ brains and transgenic mice could induce LB/LN pathology in both M83 transgenic mice (Watts et al., 2013) and wild-type mice (Masuda-Suzukake et al., 2013). It is worth noting that intracerebral injection of α-synuclein pre-formed fibrils (PFFs, misfolded α-synuclein aggregates generated from recombinant α-synuclein monomers) can also lead to a spatiotemporal accumulation and propagation of LB/LN pathology in the central nervous system, while injection of monomers does not (Luk et al., 2012; Masuda-Suzukake et al., 2013; Thakur et al., 2017). These observations strongly indicated that α-synuclein with specific conformation can serve as a complete pathogenic factor, which is different from bacteria or viruses. It also demonstrated that PFFs generated from a prokaryotic expression system have similar properties to the patient-derived fibrils in terms of pathogenicity and structure (Spillantini et al., 1998). Since both types of fibrils are formed by the self-assemble of α-synuclein monomers, the *in vitro* aggregation assay of α-synuclein PFFs can, to a certain extent, reflect the properties of pathological fibrils in the patient’s brain and reveal part of the pathogenesis of synucleinopathies.

The *in vitro* aggregation assay of α-synuclein is briefly described as follows: Recombinant α-synuclein monomers can gradually aggregate to form amyloid products through continuous shaking/agitating in a cell-free system (e.g., 5 mg/ml α-synuclein monomers in tris-buffer and agitated in a 1.5-ml EP tube at 37°C) (Narkiewicz et al., 2014). And the amount of the amyloid products can be monitored in real-time by ThT fluorescence. The classical *in vitro* aggregation assay contains three stages: (I) the initial lag phase (nucleation), when monomers assemble to form aggregation nuclei; (II) the elongation phase, when fibrils grow exponentially; (III) the stationary phase, when growth rate decreases which indicates the end of fibril formation (Wood et al., 1999). In addition, evidence shows that the fibrilization process will be accelerated in a seed-dependent pathway by adding a small number of α-synuclein seeds to the monomer solution in advance (Conway et al., 2000; Nonaka et al., 2010). The exogenous seeds can be used as pre-formed aggregated nuclei to accelerate the fibrilization rate by rapidly recruiting the monomers to the fibril ends (Shvadchak et al., 2018).

Since the fibril growth will eliminate the lag phase and directly enter the elongation phase in the presence of PFFs, shaking/agitating is not necessary for the subsequent fibril elongation. Therefore, quiescent elongation of PFFs could eliminate the influence of aggregates that formed from *de novo* aggregation under agitation. On the other hand, the quiescent incubation of fibril seeds is more in line with the mild cytosolic environment in cell soma. In this study, we explored the elongation properties of α-synuclein under a quiescent incubation condition. By using the ThT fluorescent assay, we tested several factors which could influence the ThT fluorescence curve, including seed/monomer concentration, temperature, sodium salt ion strength, surfactant, seeds pretreatment, and heterogeneous amplification of human and mouse α-synuclein. Our result further described the characteristics of α-synuclein fibril elongation under a quiescent incubation condition. This may have important implications for the *in vitro* amplification and preservation of α-synuclein aggregates to further understand the prion-like transmission mechanism of PD.

## Materials and Methods

### Expression and Purification of Human and Mouse α-Synuclein

Full-length human and mouse α-synuclein (1–140aa) proteins were expressed in *Escherichia coli* strain BL21(DE3) and purified, respectively, according to the protocol (Volpicelli-Daley et al., 2014). In brief, after the incubation overnight at 37°C with shaking, bacteria were spinning for 10 min at 6000 × *g*. Then, resuspend the pellet in high salt buffer (750 mM NaCl, 10 mM Tris, pH 7.6, 1 mM EDTA) with protease inhibitors and sonicate for a total time of 5 min (9 s on, 9 s off). Then, boil for 15 min to precipitate unwanted proteins and dialyze supernatant with 10 mM Tris, pH 7.6, 50 mM NaCl, and 1 mM EDTA. Then, concentrate protein and load onto a Superdex 200 column (Cytiva, United States). Then fractions were collected for Coomassie staining. Choose fractions with bands corresponding to the α-synuclein monomer which run slightly above the 15 kDa marker. Then, apply proteins to a Hi-Trap Q HP anion exchange column. Human or mouse α-synuclein monomer was eluted at approximately 300 mM NaCl. Then, dialyzed with 10 mM Tris, pH 7.6, and 50 mM NaCl. The monomer was aliquoted to store at –80°C or used for the fibril formation.

### Fibril Formation and Preparation of α-Synuclein Pre-form Fibrils

Purified samples of human and mouse α-synuclein monomers were concentrated at 4 mg/ml in tris-buffered saline (10 mM Tris, pH 7.6, and 50 mM NaCl). Samples were centrifuged at 100,000 × g at 4°C for 60 min to pellet aggregated material. Supernatants containing α-synuclein monomers were incubated at 37 °C with agitation (1000 rpm) in ThermoMixer (Eppendorf) for 7 days. After 7 days of agitation, the fibrils were completely formed. And these fibrillar samples were aliquoted to generate PFFs or store them at –80°C for further use. Preparation of PFFs: Human and mouse α-synuclein fibrils were diluted from 0.1 mg/ml to 1 mg/ml in tris-buffered saline (10 mM Tris, pH 7.6, and 50 mM NaCl), and sonicated for a total time of 1 min (0.5 s on, 0.5 s off). Sufficiently sonicating the large fibrils to smaller components will result in a strong LB/LN pathology. The newly generated PFFs were aliquoted to perform *in vitro* quiescent seeding assay or store at –80°C for further use. The concentration of α-synuclein monomers was determined by Nanodrop2000 at 280 nm using the theoretical extinction coefficient (ε for α-synuclein is 5960 M^–1^ cm^–1^ for human α-synuclein is 5960 and 7450 M^–1^ cm^–1^ for mouse α-synuclein).

### Negative-Stain Transmission Electron Microscopy

Negatively stained α-synuclein fibrils/PFFs for TEM were adsorbed to glow discharged 300 mesh carbon-coated copper grids (EMS) for 10 min. The grids were quickly transferred through two drops of Tris-HC1 (50 mM pH7.4) rinse, then floated upon two consecutive drops of 0.75% uranyl formate for 30 s each. The stain was either aspirated or blotted off with filter paper. Grids were allowed to dry before imaging on a Phillips CM 120 TEM operating at 100 kV.

### SDS–PAGE

Alpha-synuclein monomer and PFFs were separated using SDS–PAGE followed by incubating with commercial Coomassie Blue Fast Stain at room temperature for 20 min. Then, decolorize polyacrylamide gel with pure water for 1 h. Images were acquired under white light through a Bio-Rad instrument.

### Cell Culture and Immunocytochemistry

Primary hippocampus neuron cultures were prepared from embryonic days 16 to 18 (E16–18) in C57BL/6 mouse brains according to the previous study. All experimental procedures were performed according to the guidelines of the animal research committee of Zhujiang Hospital, Southern Medical University. Dissociated hippocampal neurons were plated onto poly-D-lysine-coated confocal dish at a density of 500,000 cells/dish. For each dish, 20 μL of 0.1 mg/ml of sufficiently sonicated PFFs will be added. The working concentration of the human or mouse PFFs was 1 ug/ml. Treated neurons were harvested for immunocytochemistry at 10 to 15 days post-PFFs treatment (17–22 DIV). To determine if the LB/LN pathologic aggregates were triggered by exogenous PFFs, the treated mouse primary hippocampus neurons were fixed with 4% paraformaldehyde/4% sucrose in PBS, followed by permeabilization with 0.1% Triton X-100. Then, neurons were stained with the monoclonal rabbit primary antibody against α-synuclein phosphorylated at S129 (1:1000; Abcam, ab51253, United States) overnight at 4°C and incubated with goat anti-rabbit IgG conjugated to Alexa Fluor 488 (1:2000, Thermo Fisher Scientific, Waltham, MA, United States) for 1 h at RT. Fluorescence images were captured by Zeiss LSM 880 (Carl Zeiss, Jena, Germany) laser-scanning microscope and analyzed by using a ZEN lite 2012 software.

### *In vitro* Quiescent Seeding Assay and ThT Fluorescence Measurement

The formation of α-synuclein aggregates was quantified by ThT dye fluorescence. This assay is based on the change in fluorescence intensity of ThT after binding with β-sheet structures from forming amyloid fibrils. The seeding assay was performed in a 48-well polypropylene PCR plate containing in each well 40 μM ThT, 1 mg/ml α-synuclein monomer, 0.1 mg/ml PFFs, and tris-buffered saline (10 mM Tris, pH 7.6, and 50 mM NaCl) up to a final volume of 5 to 10 μL. The 48-well polypropylene plate was sealed with sealing film (Axygen, Union City, CA, United States) and loaded into the eco RT-qPCR instrument (Illumina, Catalog EC-900-1001, San Diego, CA, United States) followed by incubating at 30°C to 90°C without agitation for 1 h. To correct the effects of different temperatures on ThT fluorescence intensity, fluorescence values were collected in another 10 incubation cycles at 40°C at the start and end of the incubation assay. The SYBR Green channel (Excitation: 452–486 nm, Emission: 505–545 nm) was used to detect and record the ThT fluorescence intensity during the seed incubation. After 1 h quiescent incubation, the fluorescence intensity data were exported to GraphPad Prism 9 software for processing and graphing.

## Results

### Synthetical α-Synuclein Pre-form Fibrils Induced the Lewy Body/Lewy Neurites Pathology in Mouse Primary Neuronal Cells

**FIGURE 1:**
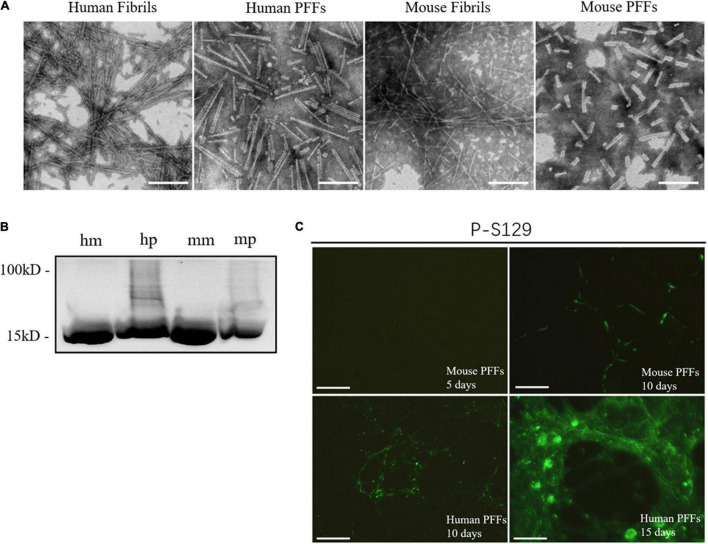
Synthetical PFFs induced the LB/LN pathology in mouse primary neuronal cells. **(A)** Typical fibril morphology of human/mouse fibrils/PFFs under the negative-stain transmission electron microscopy. **(B)** Coomassie blue staining of α-synuclein monomer and fibril seeds. **(C)** Fluorescent staining of phosphorylated α-synuclein in mouse primary neuronal cells. Time-dependent increase of phosphorylated pathological α-synuclein can be observed in human and mouse fibril seeds-treated neurons (5 days, 10 days, and 15 days, respectively). LB/LN pathology spread from the neuron axon to the cell body. After 5 days treated with mouse α-synuclein PFFs (1 μg/ml), almost no phosphorylation of pathological α-synuclein is produced, scale bar = 100 μm; Treated with mouse α-synuclein PFFs (1 μg/ml) for 10 days, a small amount of phosphorylation pathological α-synuclein can be observed, scale bar = 100 μm; After 10 days treated by human α-synuclein PFFs (1 μg/ml), a large number of phosphorylated pathological LN distributed along the axons of neurons and there was a tendency to accumulate in the cells, scale bar = 100 μm; Treated with human α-synuclein PFFs (1 μg/ml) for 15 days, a large number of round aggregates LB appeared in the neuronal soma, scale bar = 50 μm.

To ensure that the human and mouse PFFs used in the seed aggregation assay have the typical fibril morphology, we have carried out the direct morphological observations through negative-stain transmission electron microscopy (Figure 1A). TEM results clearly illustrated both human and mouse fibril structures formed by the *de novo* aggregation of monomers. For the long fibrils before sonication, their length can reach more than 1000 nm, and for the PFFs generated by sonication, their lengths are in the range of 50– 150 nm. Results show that both human and mouse PFFs formed in tris-buffered saline solution condition (50 mM NaCl 10 mM Tris PH7.6) are composed of two parallel protein fibril chains with a width of around 10 nm. This result is in line with the report that synucleinopathy strains exist in a back-to-back paired filament structure of the α-synuclein fibrilization core, including PD strains (Guerrero-Ferreira et al., 2018; Li et al., 2018) and MSA strains type I and II (Schweighauser et al., 2020). The α-synuclein monomer and PFFs were loaded to SDS-PAGE and stained with Coomassie Blue, Figure 1B. To ensure that PFFs used in the seed aggregation assay were biological pathogenic, we added these sonicated fibril seeds in mouse primary hippocampal neuron culture to test whether our PFFs can induce insoluble α-synuclein accumulation in living cells (Figure 1C). An antibody that specifically recognizes misfolded α-synuclein in the phosphorylated S129 site was used to confirm the biological pathogenicity. The immunofluorescence results show that PFFs-induced aggregates exhibited morphologies ranging from LN puncta to LB inclusions in time-dependent way. Figure 1C shows that the phosphorylated α-synuclein pathology is distributed from neuron axon to cell body with the increase of time (5 days, 10 days, and 15 days). After treating with 1 μg/ml mouse α-synuclein PFFs (mp) for 10 days, the skein-like α-synuclein LN in neuronal dendrites can be observed. After treating with 1 μg/ml of human α-synuclein PFFs (hp) for 15 days, a large number of circular LB-like inclusion can be observed in the neuron cell.

### The Effect of Seed and Monomer Concentration on Fibril Elongation Rate in Quiescent Incubation

**FIGURE 2:**
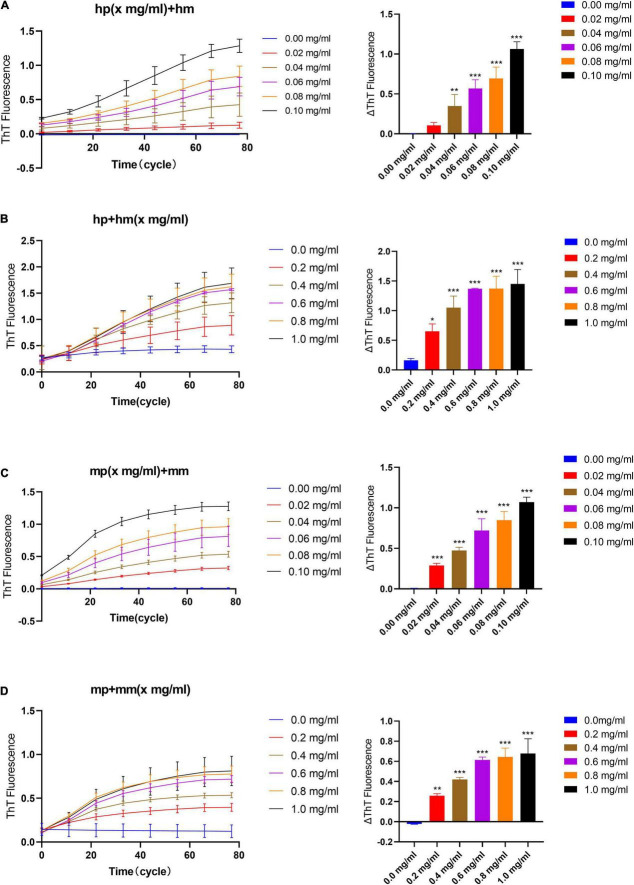
The effect of seeds and monomer concentration on the quiescent incubation curve of fibril seeds. **(A)** Fluorescence curve of quiescent incubation of human fibril seeds with different concentrations at fixed monomer concentration, left. Corresponding ΔThT fluorescence value, right. **(B)** Fluorescence curve of the quiescent incubation of human fibril seeds at different monomer concentrations, left. Corresponding ΔThT fluorescence value, right. **(C)** Fluorescence curve of quiescent incubation of mouse fibril seeds with different concentrations at fixed monomer concentration, left. Corresponding ΔThT fluorescence change value, right. **(D)** Fluorescence curve of the quiescent incubation of mouse fibril seeds at different monomer concentrations, left. Corresponding ΔThT fluorescence value, right. All data are expressed in mean ± SD. Compared with the 0 mg/ml group, *P < 0.05, **P < 0.01, ***P < 0.001.

In the quiescent incubation assay of fibril seeds, the fluorescence intensity of ThT is detected by the SYBR Green channel in the eco RT-qPCR instrument (Malisauskas et al., 2015). First, we examined the quiescent elongation rate of different concentrations of seeds with a fixed monomer concentration (1 mg/ml) at 37°C. As shown in Figure 2A, the ThT fluorescence curve always approaches to zero line when the reaction system contains only monomers but no seeds. This indicates that the α-synuclein monomer stably maintains the non-amyloid structure under quiescent incubation conditions, and the fibrillation reaction does not occur immediately. On the contrary, in the presence of seeds, the ThT fluorescence curve has increased to varying degrees with time, which indicates that fibril seeds quiescently elongated in a short time as long as there is a monomer environment around. On the other hand, in the homologous reaction of “human PFFs + human monomer (hp + hm)” or “mouse PFFs + mouse monomer (mp + mm),” the ΔThT is positively correlated with the concentration of seeds, showing that higher the concentration of seeds, faster the formation of amyloid fibrils, Figures 2A,C.

Similarly, we verified the quiescent elongation rate of different concentrations of monomer with a fixed seeds concentration, Figures 2B,D. The result shows that when the reaction system contains only seeds but no monomers, ThT fluorescence curves tend to maintain low intensity due to the presence of initial amyloid. This indicates that the α-synuclein seeds kept the amyloid fibril morphology stable under the condition of quiescent incubation for a short time, and there was no obvious fibril depolymerization or fibril generation due to seeds concentration dilution. While in the presence of different concentration monomers, the ThT fluorescence curve has risen to varying degrees, and the reaction trend is positively correlated with the increasing monomer concentration. The rising curve in the presence of certain concentration monomers indicates that the fibril elongation reaction occurs immediately and the content of amyloid fibrils in the reaction system is continuously increasing.

### The Effect of Different Incubation Temperatures on the Quiescent Incubation Curve of Fibril Seeds

**FIGURE 3:**
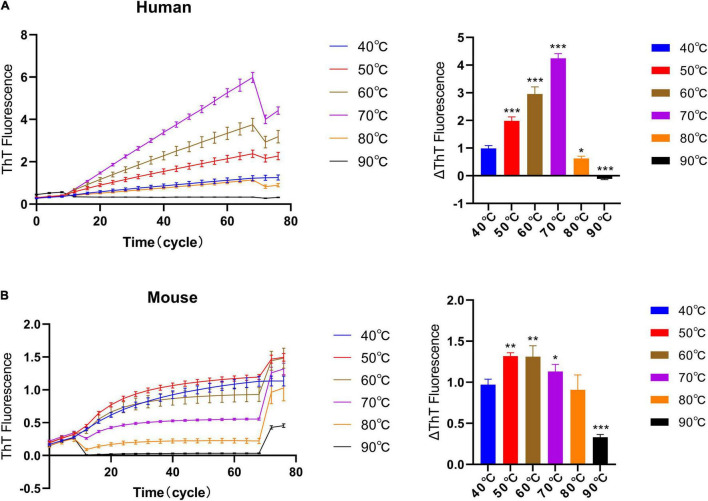
The effect of different incubation temperatures on the quiescent incubation curve of fibril seeds. Fluorescence values were collected in another 10 incubation cycles at 40°C at the start and end of the incubation assay to correct the effects of different temperatures on ThT fluorescence intensity. **(A)** Quiescent incubation curve of human α-synuclein fibril seeds + human α-synuclein monomer under different temperature conditions, left. Corresponding ΔThT fluorescence value, right. The optimal temperature for the human homologous reaction is 70°C. **(B)** Quiescent incubation curve of mouse α-synuclein fibril seeds + mouse α-synuclein monomer under different temperature conditions, left. Corresponding ΔThT fluorescence value, right. The optimal temperature for the mouse homologous reaction is 60°C. All data are expressed in mean ± SD. Compared with the 40° group, *P < 0.05, **P < 0.01, ***P < 0.001.

Next, we studied the effect of different temperatures on the ThT fluorescence curve under quiescent incubation conditions. Here, we used a reaction system with a monomer concentration of 1 mg/ml, a seed concentration of 0.1 mg/ml, and a concentration of 25 μM ThT. We tested the elongation rate of human and mouse fibril seeds under different temperature conditions to determine their optimal incubation temperature in the homologous reaction. From Figures 3A,B, we can observe that with the increase in temperature, the reaction rate of human and mouse fluorescence curves gradually increases, and then decreases after reaching the optimal temperature. The results show that the optimal temperature for quiescent incubation of “hp + hm” is 70°C and the optimal temperature for quiescent incubation of “mp + mm” is 60°C.

### The Effect of Different Solution Environments on the Quiescent Incubation Curve of Fibril Seeds

**FIGURE 4:**
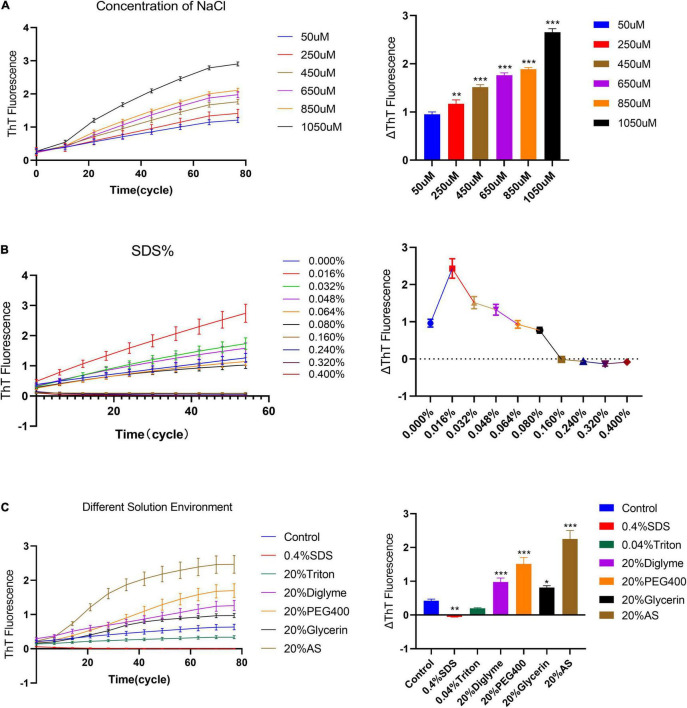
The effect of different solution environments on the seed quiescent incubation curve. **(A)** Quiescent incubation curve under different concentrations of NaCl, left. Corresponding ΔThT fluorescence value, right. ΔThT increases with increasing concentration of NaCl. Compared with the 50 μM NaCl group, **P < 0.01, ***P < 0.001. **(B)** Quiescent incubation curve under different concentrations of SDS%, left. Corresponding ΔThT fluorescence change value, right. With a lower concentration of SDS (<0.064%), the ΔThT was significantly increased. With a higher concentration of SDS (>0.064%), the ΔThT was significantly decreased. **(C)** The effects of various solution environments on the seed quiescent incubation curve, including 0.4% SDS, 0.04% Triton -X100, 20% Diglyme, 20% PEG400, 20% Glycerol, and 20% Ammonium sulfate, left. Corresponding ΔThT fluorescence change value, right. Compared with the control group, *P < 0.05, **P < 0.01, ***P < 0.001. All data are expressed in mean ± SD.

Studies have shown that the solution environment has a key effect on the fibrillation of α-synuclein (Baldwin, 1986; Buell et al., 2014). Thus, we study the elongation rate of fibril seeds in different solution environments under quiescent conditions. First, we observed the elongation rate of α-synuclein seeds under different NaCl concentrations, Figure 4A. The results show that the slope of the fluorescence curve increases with the increase of sodium ion concentration. Then, we observed the elongation rate under different SDS concentrations, Figure 4B. In the lower concentration range of SDS (<0.064%), the fluorescence curve showed a clear upward trend under the quiescent condition. The rising fluorescence curve indicates the occurrence of fibrillation reaction rather than fibril depolymerization. And as the concentration of SDS continues to increase (>0.064%), the fluorescence curve no longer rises and returns to the zero level as predicted. We also studied the effects of various solution environments on the elongation rate of human α-synuclein seeds; 0.4% Triton, 20% PEG400, 20% Dizelme, 20% Ammonium Sulfate (20%AS) were included. Figure 4C shows that the exogenous addition of 20% AS can significantly increase the upward trend of the fluorescence curve, indicating that ammonium sulfate is an obvious factor that promotes the fibril elongation reaction.

### Effect of Temperature Pretreatment of Fibril Seeds on Quiescent Incubation Curve

**FIGURE 5:**
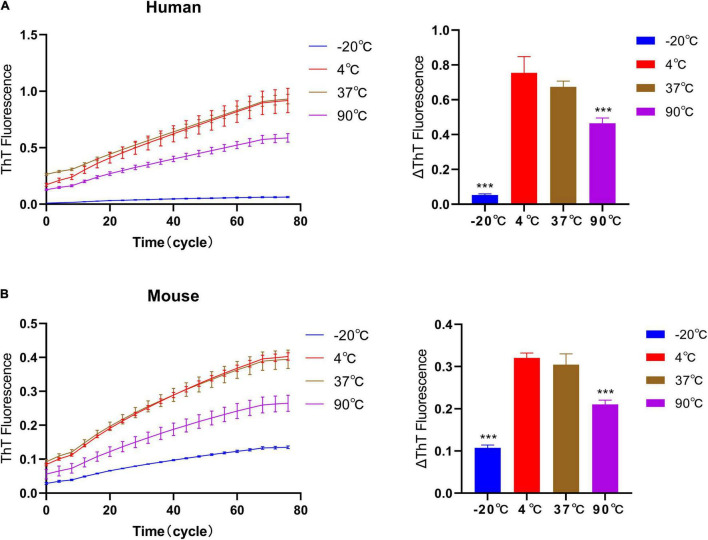
Effect of temperature pretreatment of fibril seeds on quiescent incubation curve. The human and mouse fibril seeds were pre-incubated under different temperature conditions (–20°C, 4°C, 37°C, and 90°C) for 2 h, then incubated with homologous monomers under 37°C. **(A)** The effect of temperature pretreatment of human seeds on the quiescent incubation curve, left. Corresponding ΔThT fluorescence value, right. **(B)** Effect of temperature pretreatment of mouse seeds on quiescent incubation curves, left. Corresponding ΔThT fluorescence value, right. Human and mouse α-synuclein fibril seeds still have the self-recruitment ability even after treatment at 90°C. After being treated at –20°C, the self-recruitment ability decreased significantly. All data are expressed in mean ± SD. Compared with the 37°C group, ***P < 0.001.

The quality of the exogenously added seeds is the key to establish *in vivo* seeding model of PD (Bodles et al., 2001; Bougard et al., 2016; Bongianni et al., 2017). Based on the effect of temperature on the quality of fibril seeds, we have verified the elongation rate after the temperature pretreatment of fibrils seeds, Figures 5A,B. Therefore, −20°C, 4°C, 37°C, and 90°C were used to pretreat seeds for 2 h, then verify the effect of temperature pretreatment on the self-aggregation ability of fibril seeds under 37°C. The result shows that after 2 h of pretreatment at 90°C, the fluorescence curves of human and mouse fibril seeds are lower than those in the 37°C or 4°C treatment groups, but it still showed an obvious upward trend. The upward trend of fluorescence curves suggests that fibril seeds can maintain the self-aggregation ability even under high-temperature treatment, which indicates that the pathological α-synuclein has a heat resistance similar to prion protein which cannot be inactivated by high temperature. It is worth noting that the quiescent incubation curve of human and mouse α-synuclein fibril seeds after being treated at –20°C for 2 h was significantly decreased, indicating that the fibril seeds have a decreased ability to recruit monomers.

### Quiescent Incubation Curve for Heterogeneous Amplification of Human and Mouse α-Synuclein

**FIGURE 6:**
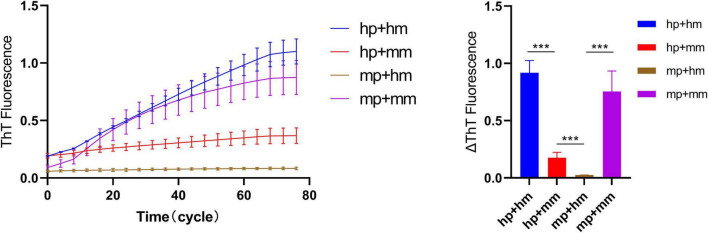
Quiescent incubation curve for heterogeneous amplification of human and mouse α-synuclein. The quiescent incubation curve for heterologous amplification, left. The concentration of human and mouse α-synuclein fibril seeds is 0.1 mg/ml and the concentration of human and mouse α-synuclein monomers is 1 mg/ml. Corresponding ΔThT fluorescence change value, right. All data are expressed in mean ± SD. ***P < 0.001.

Heterologous amplification (Cross seeding) is an interesting feature of α-synuclein strains. The quiescent incubation assay was used to explore the rate of heterogeneous amplification of human and mouse α-synuclein (“hp + mm” and “mp + hm”). Figure 6 shows that during the elongation reaction of “hp + mm,” the fluorescence curve increased significantly, indicating the increase in the amount of amyloid in the reaction system, and further explaining the mouse α-synuclein monomers can be recruited by human α-synuclein seeds, which also verifies the pathological recruitability of human fibril seeds in treating with mouse primary neurons. During the elongation reaction of “mp + hm,” the fluorescence curve increases slightly in a short period of time, indicating that the mouse α-synuclein seeds have a weaker ability to recruit human monomer.

## Discussion

Parkinson’s disease is considered to be a neurodegenerative disease determined by factors, such as environment, age, and genes. Increasing evidence shows that injection of synthetic pre-form fibrils into the central nervous system or periphery tissue can induce LB/LN pathology in transgene or non-transgene mice, which indicates that the onset of PD is probably related to the formation of the initial misfolded protein. Therefore, α-synuclein in aggregate form has been refocused as a prion-like protein factor in recent years and abnormal deposition of α-synuclein in multiple tissues is considered to be the most important biomarker of PD. *In vitro* aggregation assay can reflect the self-assembly ability of α-synuclein aggregates. This may have important implications for understanding prion-like spreading mechanism of α-synuclein aggregates.

In this study, we explored the effects of different solution environments on the elongation rate of α-synuclein fibril seeds under quiescent conditions. Alpha-synuclein can aggregate in various solution environments to form different strains, which have diverse characteristics in the fibrillation process, protein structure, and biological pathogenicity. NaCl ion intensity was one of the main factors contributing to the formation of two different strains (Bousset et al., 2013; Peelaerts et al., 2015). Our results demonstrate that higher salt ion concentration or exogenous addition of ammonium sulfate may promote the process of fibrillation to some extent. The discovery of the pro-fibrilization solution environment may help optimize RT-QuiC/PMCA assay that has been recently used in ultra-micro detecting α-synuclein aggregates in the body fluid or skin tissue of PD patients. In addition, SDS is a strong anionic detergent which can unravel the complex protein structure and depolymerize the polymerized α-synuclein fibrils. Therefore, SDS should play an inhibitory role in the reaction system. But interestingly, our results demonstrate that lower concentrations of SDS (<0.064%) can promote fibril elongation, while higher concentrations of SDS (>0.064%) can inhibit fibril elongation. It is consistent with the finding that sub-micellar concentrations of SDS will promote fibrillation of α-synuclein by rapid formation of small clusters of α-synuclein around shared micelles, although the morphology of SDS-induced fibril is totally different from tris-buffer saline, which presents a flexible worm-like appearance (Giehm et al., 2010, 2011).

The results of our concentration experiment indicate that quiescent incubation assay is also suitable for *in vitro* amplification of amyloid fibril in a certain concentration range. It is consistent with agitation incubation in PMCA/RT-QuiC assay. PMCA or RT-QuiC assays usually use 1/2*t* (average lag time *t*, the time corresponding to 1/2 the maximum fluorescence intensity) to illustrate the speed of the total fibrillation reaction rate. The higher the seed concentration, the smaller the 1/2*t* value (Fairfoul et al., 2016; Shahnawaz et al., 2017; Becker et al., 2018). In addition, the results of heterogeneous amplification of human and mouse α-synuclein showed that human fibril seeds can cross seed mouse monomers in a cell-free system. This heterogeneous recruitment ability may be the main mechanism by which human-derived PFFs can induce LB prion-like transmission pathology in mouse models. On the other hand, we found that the amplification trend of heterogeneous human and mouse α-synuclein is also affected by the solution environment, for example, in 20% ammonium sulfate (data are not given). Therefore, different strains may be determined by both strains themselves and the solution environment.

Previous studies have shown that reduced recruitability of fibril seeds can lead to the reduction of LB pathology and cytotoxicity, suggesting that recruitability of α-synuclein is the basis of its transmissibility and cytotoxicity. For example, after the core peptide of NAC was knocked out, the recruitment/self-aggregation ability of α-synuclein was lost, followed by the failure of fibril formation, which could not induce LB pathology (Giasson et al., 2001; Luk et al., 2009; Sacino et al., 2014). Conversely, A53T or E46K mutations predispose monomers to aggregate to form fibrils, leading to a stronger LB pathology in mouse models. In SNCA knockout mice, the addition of exogenous PFFs did not induce LB pathology and PD symptoms due to the lack of endogenous monomers. In our study, the upward trend of the ThT curve indicates that the fibril elongation in the reaction system is in progress, and the slope of the curve and the ΔThT reflect the recruitability of fibril seeds in the current solution environment. Our temperature pretreatment results demonstrate that the elongation rate of the fibril seeds can be reduced after low-temperature pretreatment. One important reason may be that low-temperature treatment depolymerizes fibril seeds by reducing the hydrophobic bond effect, thus reducing the overall recruitability. Therefore, it is not recommended to store biological samples with α-synuclein aggregates at –20°C.

It seems that the onset of PD and the spread of LB pathology are related to the nucleation and elongation stages in the α-synuclein fibrilization process, respectively. The abnormal initiation of the fibril nucleation stage may be involved in the formation of early pathological fibril seeds in cell soma, while the elongation stage of fibril seeds is mainly related to the transcellular, long-distance, prion-like transmission of existing LB pathology. In our study, we investigated α-synuclein fibrilization under quiescent conditions. It subtracted the nucleation stage from *de novo* fibrilization. In addition, the quiescent condition is closer to the mild environment of the intracellular fluid, and it avoids the oscillating shear forces and reduces the air–water interfacial area. Therefore, the spreading characteristics of PD can be more accurately reflected through the quiescent condition.

## Conclusion

By using the ThT fluorescence assay, we compared and quantified the elongation fluorescence curves to explore the factors that affect fibril elongation. These factors include proteins’ concentration, temperature, NaCl strength, SDS, temperature pretreatment, and so on. Our work further described the characteristics of α-synuclein fibril elongation under a quiescent incubation condition. This may have important implications for the *in vitro* amplification and preservation of α-synuclein aggregates to further understand the prion-like transmission mechanism of PD.

## Data Availability Statement

The original contributions presented in the study are included in the article/supplementary material, further inquiries can be directed to the corresponding author.

## Ethics Statement

The animal study was reviewed and approved by Ethics Committee and the Care of the Animals of Southern Medical University of China.

## Author Contributions

HM and SZ designed the study. HM and YY contributed to experimental implementation. HM, CQ, BW, and LX contributed to data analysis. HM and XS contributed to manuscript writing. All authors approved the final version of the manuscript.

## Conflict of Interest

The authors declare that the research was conducted in the absence of any commercial or financial relationships that could be construed as a potential conflict of interest.

## Publisher’s Note

All claims expressed in this article are solely those of the authors and do not necessarily represent those of their affiliated organizations, or those of the publisher, the editors and the reviewers. Any product that may be evaluated in this article, or claim that may be made by its manufacturer, is not guaranteed or endorsed by the publisher.

## Abbreviations

PFFs: α-synuclein pre-form fibrils
ThT: Thioflavin T
PMCA: protein misfold cycle amplification
RT-QuiC: real-time quaking-induced conversion
LB: Lewy Body
LN: Lewy Neurites
NAC: non-A beta component of Alzheimer’s disease amyloid
hm: human α-synuclein monomers
hp: human α-synuclein pre-form fibrils
mm: mouse α-synuclein monomers
mp: mouse α-synuclein pre-form fibrils.

## References

[B1] Baldwin R. L. (1986). Temperature dependence of the hydrophobic interaction in protein folding. *Proc. Natl. Acad. Sci. U.S.A.* 83 8069–8072. 10.1073/pnas.83.21.8069 3464944 PMC386868

[B2] Becker K. Wang X. Vander Stel K. Chu Y. Kordower J. Ma J. (2018). Detecting alpha synuclein seeding activity in formaldehyde-fixed MSA patient tissue by PMCA. *Mol. Neurobiol.* 55 8728–8737. 10.1007/s12035-018-1007-y 29589283 PMC6153717

[B3] Bodles A. M. Guthrie D. J. Greer B. Irvine G. B. (2001). Identification of the region of non-Aβ component (NAC) of alzheimer’s disease amyloid responsible for its aggregation and toxicity. *J. Neurochem.* 78 384–395. 10.1046/j.1471-4159.2001.00408.x 11461974

[B4] Bongianni M. Orrù C. Groveman B. R. Sacchetto L. Fiorini M. Tonoli G. (2017). Diagnosis of human prion disease using real-time quaking-induced conversion testing of olfactory mucosa and cerebrospinal fluid samples. *JAMA Neurol.* 74 155–162. 10.1001/jamaneurol.2016.4614 27942718

[B5] Bougard D. Brandel J. P. Bélondrade M. Béringue V. Segarra C. Fleury H. (2016). Detection of prions in the plasma of presymptomatic and symptomatic patients with variant creutzfeldt-jakob disease. *Sci. Transl. Med.* 8:370ra182. 10.1126/scitranslmed.aag1257 28003547

[B6] Bousset L. Pieri L. Ruiz-Arlandis G. Gath J. Jensen P. H. Habenstein B. (2013). Structural and functional characterization of two alpha-synuclein strains. *Nat. Commun.* 4:2575. 10.1038/ncomms3575 24108358 PMC3826637

[B7] Buell A. K. Galvagnion C. Gaspar R. Sparr E. Vendruscolo M. Knowles T. P. (2014). Solution conditions determine the relative importance of nucleation and growth processes in α-synuclein aggregation. *Proc. Natl. Acad. Sci. U S A.* 111 7671–7676. 10.1073/pnas.1315346111 24817693 PMC4040554

[B8] Conway K. A. Lee S. J. Rochet J. C. Ding T. T. Williamson R. E. Lansbury P. T. Jr. (2000). Acceleration of oligomerization, not fibrillization, is a shared property of both alpha-synuclein mutations linked to early-onset parkinson’s disease: implications for pathogenesis and therapy. *Proc. Natl. Acad. Sci. U.S.A.* 97 571–576. 10.1073/pnas.97.2.571 10639120 PMC15371

[B9] Fairfoul G. McGuire L. I. Pal S. Ironside J. W. Neumann J. Christie S. (2016). Alpha-synuclein RT-Qu IC in the CSF of patients with alpha-synucleinopathies. *Ann. Clin. Transl. Neurol.* 3 812–818. 10.1002/acn3.338 27752516 PMC5048391

[B10] Giasson B. I. Murray I. V. Trojanowski J. Q. Lee V. M.-Y. (2001). A hydrophobic stretch of 12 amino acid residues in the middle of α-synuclein is essential for filament assembly. *J. Biol. Chem.* 276 2380–2386. 10.1074/jbc.M008919200 11060312

[B11] Giehm L. Lorenzen N. Otzen D. E. (2011). Assays for alpha-synuclein aggregation. *Methods* 53 295–305. 10.1016/j.ymeth.2010.12.008 21163351

[B12] Giehm L. Oliveira C. L. Christiansen G. Pedersen J. S. Otzen D. E. (2010). SDS-induced fibrillation of alpha-synuclein: an alternative fibrillation pathway. *J. Mol. Biol.* 401 115–133. 10.1016/j.jmb.2010.05.060 20540950

[B13] Guerrero-Ferreira R. Taylor N. M. Mona D. Ringler P. Lauer M. E. Riek R. (2018). Cryo-EM structure of alpha-synuclein fibrils. *Elife* 7:e36402. 10.7554/eLife.36402 29969391 PMC6092118

[B14] Hu D. Sun X. Liao X. Zhang X. Zarabi S. Schimmer A. (2019). Alpha-synuclein suppresses mitochondrial protease ClpP to trigger mitochondrial oxidative damage and neurotoxicity. *Acta. Neuropathol.* 137 939–960. 10.1007/s00401-019-01993-2 30877431 PMC6531426

[B15] Lee H. J. Khoshaghideh F. Patel S. Lee S. J. (2004). Clearance of alpha-synuclein oligomeric intermediates via the lysosomal degradation pathway. *J. Neurosci.* 24 1888–1896. 10.1523/jneurosci.3809-03.2004 14985429 PMC6730405

[B16] Li B. Ge P. Murray K. A. Sheth P. Zhang M. Nair G. (2018). Cryo-EM of full-length alpha-synuclein reveals fibril polymorphs with a common structural kernel. *Nat. Commun.* 9:3609. 10.1038/s41467-018-05971-2 30190461 PMC6127345

[B17] Lindersson E. Beedholm R. Højrup P. Moos T. Gai W. Hendil K. B. (2004). Proteasomal inhibition by alpha-synuclein filaments and oligomers. *J. Biol. Chem.* 279 12924–12934. 10.1074/jbc.M306390200 14711827

[B18] Luk K. C. Kehm V. M. Zhang B. O’Brien P. Trojanowski J. Q. Lee V. M. (2012). Intracerebral inoculation of pathological α-synuclein initiates a rapidly progressive neurodegenerative α-synucleinopathy in mice. *J. Exp. Med.* 209 975–986. 10.1084/jem.20112457 22508839 PMC3348112

[B19] Luk K. C. Song C. O’Brien P. Stieber A. Branch J. R. Brunden K. R. (2009). Exogenous α-synuclein fibrils seed the formation of lewy body-like intracellular inclusions in cultured cells. *Proc. Natl. Acad. Sci. U.S.A.* 106 20051–20056. 10.1073/pnas.0908005106 19892735 PMC2785290

[B20] Malisauskas R. Botyriute A. Cannon J. G. Smirnovas V. (2015). Flavone derivatives as inhibitors of insulin amyloid-like fibril formation. *PLoS One* 10:e0121231. 10.1371/journal.pone.0121231 25799281 PMC4370379

[B21] Masuda-Suzukake M. Nonaka T. Hosokawa M. Oikawa T. Arai T. Akiyama H. (2013). Prion-like spreading of pathological α-synuclein in brain. *Brain* 136 1128–1138. 10.1093/brain/awt037 23466394 PMC3613715

[B22] Narkiewicz J. Giachin G. Legname G. (2014). In vitro aggregation assays for the characterization of α-synuclein prion-like properties. *Prion* 8 19–32. 10.4161/pri.28125 24552879 PMC4116381

[B23] Nonaka T. Watanabe S. T. Iwatsubo T. Hasegawa M. (2010). Seeded aggregation and toxicity of {alpha}-synuclein and tau: cellular models of neurodegenerative diseases. *J. Biol. Chem.* 285 34885–34898. 10.1074/jbc.M110.148460 20805224 PMC2966103

[B24] Peelaerts W. Bousset L. Van der Perren A. Moskalyuk A. Pulizzi R. Giugliano M. (2015). α-Synuclein strains cause distinct synucleinopathies after local and systemic administration. *Nature* 522 340–344. 10.1038/nature14547 26061766

[B25] Sacino A. N. Brooks M. Thomas M. A. McKinney A. B. Lee S. Regenhardt R. W. (2014). Intramuscular injection of α-synuclein induces CNS α-synuclein pathology and a rapid-onset motor phenotype in transgenic mice. *Proc. Natl. Acad. Sci. U.S.A.* 111 10732–10737. 10.1073/pnas.1321785111 25002524 PMC4115570

[B26] Schweighauser M. Shi Y. Tarutani A. Kametani F. Murzin A. G. Ghetti B. (2020). Structures of α-synuclein filaments from multiple system atrophy. *Nature* 585 464–469. 10.1038/s41586-020-2317-6 32461689 PMC7116528

[B27] Shahnawaz M. Tokuda T. Waragai M. Mendez N. Ishii R. Trenkwalder C. (2017). Development of a biochemical diagnosis of Parkinson disease by detection of α-synuclein misfolded aggregates in cerebrospinal fluid. *JAMA Neurol.* 74 163–172. 10.1001/jamaneurol.2016.4547 27918765

[B28] Shvadchak V. V. Afitska K. Yushchenko D. A. (2018). Inhibition of α−synuclein amyloid fibril elongation by blocking fibril ends. *Angew. Chem. Int. Ed.* 57 5690–5694. 10.1002/anie.201801071 29575453

[B29] Spillantini M. G. Crowther R. A. Jakes R. Hasegawa M. Goedert M. (1998). α-Synuclein in filamentous inclusions of lewy bodies from parkinson’s disease and dementia with lewy bodies. *Proc. Natl. Acad. Sci.* 95 6469–6473. 10.1073/pnas.95.11.6469 9600990 PMC27806

[B30] Spillantini M. G. Schmidt M. L. Lee V. M. Trojanowski J. Q. Jakes R. Goedert M. (1997). Alpha-synuclein in lewy bodies. *Nature* 388 839–840. 9278044 10.1038/42166

[B31] Thakur P. Breger L. S. Lundblad M. Wan O. W. Mattsson B. Luk K. C. (2017). Modeling Parkinson’s disease pathology by combination of fibril seeds and α-synuclein overexpression in the rat brain. *Proc. Natl. Acad. Sci. U.S.A.* 114 E8284–E8293. 10.1073/pnas.1710442114 28900002 PMC5625925

[B32] Volpicelli-Daley L. A. Luk K. C. Lee V. M. (2014). Addition of exogenous α-synuclein preformed fibrils to primary neuronal cultures to seed recruitment of endogenous α-synuclein to Lewy body and lewy neurite–like aggregates. *Nat. protoco.* 9:2135. 10.1038/nprot.2014.143 25122523 PMC4372899

[B33] Wang X. Becker K. Levine N. Zhang M. Lieberman A. P. Moore D. J. (2019). Pathogenic alpha-synuclein aggregates preferentially bind to mitochondria and affect cellular respiration. *Acta. Neuropathol. Commun.* 7:41. 10.1186/s40478-019-0696-4 30871620 PMC6419482

[B34] Watts J. C. Giles K. Oehler A. Middleton L. Dexter D. T. Gentleman S. M. (2013). Transmission of multiple system atrophy prions to transgenic mice. *Proc. Natl. Acad. Sci. U.S.A.* 110 19555–19560. 10.1073/pnas.1318268110 24218576 PMC3845125

[B35] Winner B. Jappelli R. Maji S. K. Desplats P. A. Boyer L. Aigner S. (2011). In vivo demonstration that alpha-synuclein oligomers are toxic. *Proc. Natl. Acad. Sci. U.S.A.* 108 4194–4199. 10.1073/pnas.1100976108 21325059 PMC3053976

[B36] Wood S. J. Wypych J. Steavenson S. Louis J. C. Citron M. Biere A. L. (1999). α-Synuclein fibrillogenesis is nucleation-dependent implications for the pathogenesis of Parkinson’ s disease. *J. Biol. Chem.* 274 19509–19512. 10.1074/jbc.274.28.19509 10391881

